# Mucosal-Associated Invariant T (MAIT) Cells Are Highly Activated and Functionally Impaired in COVID-19 Patients

**DOI:** 10.3390/v13020241

**Published:** 2021-02-03

**Authors:** Sebastian Deschler, Juliane Kager, Johanna Erber, Lisa Fricke, Plamena Koyumdzhieva, Alexandra Georgieva, Tobias Lahmer, Johannes R. Wiessner, Florian Voit, Jochen Schneider, Julia Horstmann, Roman Iakoubov, Matthias Treiber, Christof Winter, Jürgen Ruland, Dirk H. Busch, Percy A. Knolle, Ulrike Protzer, Christoph D. Spinner, Roland M. Schmid, Michael Quante, Katrin Böttcher

**Affiliations:** 1 Department of Internal Medicine II, University Hospital Rechts der Isar, School of Medicine, Technical University of Munich (TUM), 81675 Munich, Germany; s.deschler@tum.de (S.D.); Juliane.Kager@mri.tum.de (J.K.); Johanna.Erber@mri.tum.de (J.E.); Lisa.Fricke@mri.tum.de (L.F.); Plamena.Koyumdzhieva@mri.tum.de (P.K.); alexandra.georgieva@campus.lmu.de (A.G.); Tobias.Lahmer@mri.tum.de (T.L.); JohannesRoman.Wiessner@mri.tum.de (J.R.W.); Florian.Voit@mri.tum.de (F.V.); Jochen.Schneider@mri.tum.de (J.S.); Julia.Horstmann@mri.tum.de (J.H.); Roman.Iakoubov@mri.tum.de (R.I.); Matthias.treiber@mri.tum.de (M.T.); christoph.spinner@mri.tum.de (C.D.S.); RolandM.Schmid@mri.tum.de (R.M.S.); michael.quante@uniklinik-freiburg.de (M.Q.); 2 Institute of Clinical Chemistry and Pathobiochemistry, School of Medicine, Technical University of Munich, 81675 Munich, Germany; Christof.winter@tum.de (C.W.); j.ruland@tum.de (J.R.); 3 German Cancer Consortium (DKTK), German Cancer Research Center (DKFZ), 69120 Heidelberg, Germany; 4 Institute for Medical Microbiology, Immunology and Hygiene, Technical University of Munich, 81675 Munich, Germany; dirk.busch@mri.tum.de; 5 Institute of Molecular Immunology and Experimental Oncology, University Hospital Rechts der Isar, Technical University of Munich, 81675 Munich, Germany; percy.knolle@tum.de; 6 Institute of Virology, Technical University of Munich/Helmholtz Zentrum München, 81675 Munich, Germany; protzer@tum.de; 7 German Center for Infection Research (DZIF), 38124 Braunschweig, Partner Site Munich, Germany; 8 Freiburg University Medical Center, Department of Medicine II (Gastroenterology, Hepatology, Endocrinology and Infectious Diseases), Faculty of Medicine, University of Freiburg, 79106 Freiburg, Germany

**Keywords:** COVID-19, SARS-CoV-2, mucosal-associated invariant T (MAIT) cells

## Abstract

Coronavirus disease 2019 (COVID-19), caused by infection with severe acute respiratory syndrome coronavirus 2 (SARS-CoV-2), comprises mild courses of disease as well as progression to severe disease, characterised by lung and other organ failure. The immune system is considered to play a crucial role for the pathogenesis of COVID-19, although especially the contribution of innate-like T cells remains poorly understood. Here, we analysed the phenotype and function of mucosal-associated invariant T (MAIT) cells, innate-like T cells with potent antimicrobial effector function, in patients with mild and severe COVID-19 by multicolour flow cytometry. Our data indicate that MAIT cells are highly activated in patients with COVID-19, irrespective of the course of disease, and express high levels of proinflammatory cytokines such as IL-17A and TNFα ex vivo. Of note, expression of the activation marker HLA-DR positively correlated with SAPS II score, a measure of disease severity. Upon MAIT cell-specific in vitro stimulation, MAIT cells however failed to upregulate expression of the cytokines IL-17A and TNFα, as well as cytolytic proteins, that is, granzyme B and perforin. Thus, our data point towards an altered cytokine expression profile alongside an impaired antibacterial and antiviral function of MAIT cells in COVID-19 and thereby contribute to the understanding of COVID-19 immunopathogenesis.

## 1. Introduction

Infection with severe acute respiratory syndrome coronavirus 2 (SARS-CoV-2) causing coronavirus disease 2019 (COVID-19) has resulted in a global pandemic with a high number of fatalities [1,2]. As of December 2020, the World Health Organisation (WHO) had accounted for over 70 million cases and over 1.6 million deaths worldwide, with case numbers still on the rise. Whereas about 80% of COVID-19 patients only experience mild symptoms such as fatigue, fever and dry cough, a severe course of disease occurs in a small subgroup of approximately 5% of COVID-19 patients [3,4,5]. These patients develop acute respiratory distress syndrome (ARDS) with respiratory failure, as well as systemic inflammatory response syndrome (SIRS) and multi-organ failure. In contrast to a 2.3% overall mortality rate in COVID-19 patients [3], a mortality of more than 50% has been reported for patients with severe COVID-19 [6]. It has been proposed that the host’s immune system plays a pivotal role for the determination of the course of disease in COVID-19. While activation of the immune system is needed to eliminate SARS-CoV-2 in the early stage of disease, an excessive immune response can lead to systemic hyperinflammation, severe pulmonary damage and ARDS, requiring intensive care treatment and invasive ventilation [7]. ARDS iscommonly caused by direct pulmonary damage induced by viral, bacterial or fungal infection, but also occurs in sterile systemic inflammation, that is, in the absence of infection. The immunopathogenesis of ARDS is multifactorial, but has been shown to involve both the innate and adaptive immune system, which can trigger pulmonary damage through various mechanisms (reviewed in [8]). In COVID-19, the adaptive immune system is crucial for elimination of SARS-CoV-2, generating antiviral humoral and cellular responses through antibody-producing B cells and cytotoxic effector T lymphocytes [9,10]. Nevertheless, activation of the innate immune system, which can prompt rapid, first-line, antigen-independent immune responses, is thought to play a pivotal role for the pathogenesis of COVID-19 and its complications, too. Mucosal-associated invariant T (MAIT) cells are innate-like T cells recognising bacterial-derived vitamin B metabolites as antigens [11]. Besides circulating in peripheral blood, MAIT cells are particularly enriched at mucosal surfaces such as the lungs [12,13]. Upon activation, MAIT cells express the pro-inflammatory cytokines IFNγ, TNFα and IL-17A. Moreover, they can eliminate bacterially infected cells by secretion of cytolytic proteins [14,15,16,17]. Hence, MAIT cells have been shown to protect against pulmonary infection with various bacteria (reviewed in [18]) and to inflict IL-17A-mediated inflammation in pulmonary infection [19]. Importantly, although MAIT cells cannot recognise viral antigen, they have recently been proposed to express antiviral properties. Thus, MAIT cells are highly activated in patients with influenza and dengue virus infection [20]. Activation of MAIT cells in viral infections is antigen-independent and mediated by the cytokines IL-12 and IL-18, rendering MAIT cells capable of orchestrating inflammation even in the absence of bacterial infection [20,21]. Along these lines, MAIT cells have been shown to protect against a lethal course of influenza in a murine model [22]. Up until now, it remains unclear how MAIT cells contribute to the immunopathogenesis of COVID-19. We have therefore analysed MAIT cells in a cohort of 43 COVID-19 patients admitted to the University hospital rechts der Isar, Munich, Germany with either mild or severe course of COVID-19. Our data show that MAIT cells express a highly activated phenotype in peripheral blood of COVID-19 patients, irrespective of the course of disease. Moreover, MAIT cells from COVID-19 patients show an altered cytokine expression profile and fail to upregulate expression of cytotoxic molecules upon in vitro stimulation. Taken together, our data indicate severe alterations of MAIT cells in COVID-19 patients, which may contribute to the pathogenesis of COVID-19 and its complications.

## 2. Materials and Methods

### 2.1. Primary Cell Isolation

All blood samples were obtained from patients or healthy volunteers at the Klinikum Rechts der Isar after giving informed consent (see Table 1 for detailed patient characteristics). Peripheral blood mononuclear cells (PBMCs) were isolated by gradient centrifugation using Pancoll human gradient solution (PAN-Biotech, Aidenbach, Germany), cryopreserved and stored at −80 °C until analysis.

### 2.2. Flow Cytometric Analysis

Cells were stained using the following antibodies:

Surface markers: CD3 BV605 (OKT3), CD3 PE-Cy7 (HIT3a), CD4 Pacific Blue (SK3), CD8 AF700 (SK1), CD8 APC (SK1), CD16 APC-Cy7 (3G8), CD16 PE-Cy7 (3G8), CD19 PE dazzle 594 (HIB19), CD25 APC (M-A251), CD38 PE dazzle 594 (HIT2), CD45RO PE-Cy7 (UCHL1), CD56 AF700 (5.1H11), CD56 PE-Cy7 (5.1H11), CD62L BV421 (DREG-56), CD69 PE dazzle 594 (FN50), CD127 FITC (A019D5), CD161 BV605 (HP-3G10), CD218a (IL-18Ra) PE (H44), CD279 (PD1) PE (EH12.2H7), TCR Vα7.2 BV421 (3C10), TCR Vδ2 FITC (B6) (Biolegend, San Diego, CA, USA), CD4 PE CF594 (RPA-T4), CD8 FITC (HIT8a), CD152 (CTLA-4) BV 786 (BNI3), CD212 (IL-12Rβ2) APC (2.4E6), HLA-DR FITC (G46-6), CD161 APC (191B8) (Miltenyi Biotec, Bergisch Gladbach, Germany).

Intracellular markers: IFNγ APC-Cy7 (4S.B3), IL-17A PerCP-Cy5.5 (BL168), TNFα PE (MaB11) (Biolegend, San Diego, CA, USA), Granzyme B AF700 (GB11), Perforin AF488 (δG9) (BD Bioscience, San Jose, CA, USA), IFNγ FITC (45-15) (Miltenyi Biotec, Bergisch Gladbach, Germany).

Dead cells were excluded using live/dead fixable aqua dead cell stain kit (Thermo Fisher, Waltham, MA, USA). For staining of MAIT cells, human MR1 5-OP-RU BV421-labeled tetramer was employed as described before [23]. Tetramer technology was developed jointly by Dr. James McCluskey, Dr. Jamie Rossjohn and Dr. David Fairlie, and the material was produced by the NIH Tetramer Core Facility as permitted to be distributed by the University of Melbourne. For staining of intracellular markers, cells were fixed and permeabilized using Intracellular Fixation and Permeabilisation Buffer Set (Thermo Fisher, Waltham, MA, USA). Samples were acquired with SP6800 Spectral Analyzer (Sony, Tokyo, Japan) and analysed with FlowJo 10.7 (BD Biosciences, San Jose, CA, USA).

### 2.3. In Vitro Stimulation Assays

Freshly thawed PBMCs were stimulated in vitro with IL-12 (50 ng/mL) (Miltenyi Biotec, Bergisch Gladbach, Germany) and IL-18 (50 ng/mL) (R&D systems, Minneapolis, MN, USA), or Escherichia coli (E. coli) (TOP10, Thermo Fisher, Waltham, MA, USA) for 24 h as described before [21]. Briefly, E. coli were fixed in 4% paraformaldehyde for 30 min at room temperature, washed extensively and added to PBMCs at 10 bacteria per cell for 24 h. Brefeldin A and Monensin (Thermo Fisher) were added for the last 4 h of culture when intracellular cytokine expression was analysed by flow cytometry.

### 2.4. Statistical Analysis

All graphs and statistical analysis were completed using Prism software version 9 (Graph Pad, San Diego, CA, USA). Values are expressed as mean ± standard error of the mean (SEM), and statistical significance was analysed with the appropriate test as indicated in the figure legends.

## 3. Results

### 3.1. The Frequency of Adaptive Immune Cell Populations Is Altered in Patients with COVID-19, Irrespective of the Clinical Course of Disease

**Figure 1:**
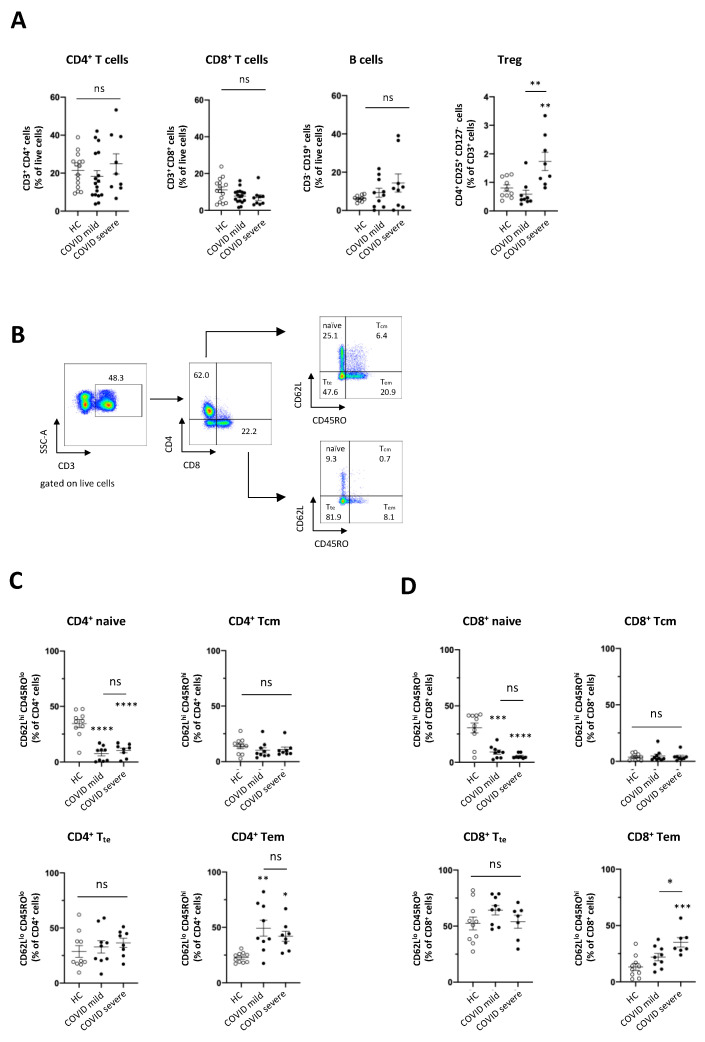
Alterations in adaptive immune cell frequency in COVID-19 patients. (**A**) Frequency of conventional T cells (HC *n* = 15; COVID mild *n* = 17; COVID severe *n* = 9), B cells (HC *n* = 15; COVID mild *n* = 10; COVID severe *n* = 9) and Treg cells (HC *n* = 10; COVID mild *n* = 9; COVID severe *n* = 8) in peripheral blood of COVID-19 patients and healthy controls (HC); (**B**) Flow cytometry gating strategy for identification of T cell subsets; Frequency of CD4^+^ (**C**) and CD8^+^ (**D**) T cell subsets in peripheral blood of COVID-19 patients and healthy controls (HC *n* = 10; COVID mild *n* = 9; COVID severe *n* = 8). Data are presented as mean ± SEM and were pooled from three independent experiments; each symbol represents one patient; ** *p* < 0.01, *** *p* < 0.001, **** *p* < 0.0001 vs. HC or as indicated, data were assessed using one-way analysis of variance (ANOVA) with Tukey’s multiple comparisons test, ns = not significant; T_cm_= central memory T cells, T_em_ = effector memory T cells and T_te_ = terminally differentiated T effector cells.

The immune system has been proposed to play an important role for the pathogenesis of COVID-19 [7,24,25]. To dissect alterations of the immune system in patients with COVID-19, we analysed subsets of adaptive and innate immune cells using multicolour flow cytometry in a cohort of 43 hospitalised COVID-19 patients with either mild (22 patients, 51%) or severe (21 patients, 49%) clinical course, and compared them to 25 healthy volunteers (see Table 1 for detailed characteristic). Patients with mild COVID-19 were treated on a general infectious diseases ward, whereas all patients with severe COVID-19 were admitted to intensive care unit (ICU). Oxygen therapy <15L O_2_ was required by 27% of patients with mild COVID-19. Amongst the 21 patients with severe COVID-19, 19 patients (90%) required invasive ventilation, of which 5 were treated with additional extracorporeal membrane oxygenation (ECMO) (Table 1). We first analysed the frequency of conventional CD4^+^ and CD8^+^ T cells, as wells as B cells and CD4^+^CD25^+^CD127^−^ regulatory T cells (Treg, see gating strategy in Figure S1A) in a PBMC pool isolated from peripheral blood of COVID-19 patients with mild and severe course of disease. As shown in Figure 1A, we did not observe significant differences in total CD4^+^ T cell, CD8^+^ T cell or B cell frequency in peripheral blood of COVID-19 patients compared to healthy controls, irrespective of disease severity. In contrast, Treg frequency was significantly increased in patients with severe COVID-19, compared to healthy controls. Interestingly, Treg frequency in patients with severe COVID-19 was also significantly increased in comparison to patients with mild COVID-19 (Figure 1A). The use of multicolour flow cytometry further allowed to define subsets of adaptive T cells by expression of CD45RO and CD62L (see Figure 1B for gating strategy) as naïve, central memory (T_cm_), effector memory (T_em_) and terminally differentiated effector T cells (T_te_). We observed a significant decline of naïve CD4^+^ and CD8^+^ T cells in patients with both mild and severe COVID-19. Moreover, CD8^+^ T_em_ cells were significantly increased in patients with severe COVID-19, and CD4^+^ T_em_ cells were significantly increased in COVID-19 patients with either mild or severe COVID-19. In contrast, CD4^+^ and CD8^+^ T_cm_ cell, as well as T_te_ frequency was unchanged in COVID-19 patients irrespective of the course of disease (Figure 1C,D). These data indicate changes in the adaptive immune system pointing towards activation of an adaptive immune response in patients with COVID-19.

### 3.2. MAIT Cells Are Severely Reduced and Phenotypically Altered in Peripheral Blood of Patients with COVID-19

**Figure 2:**
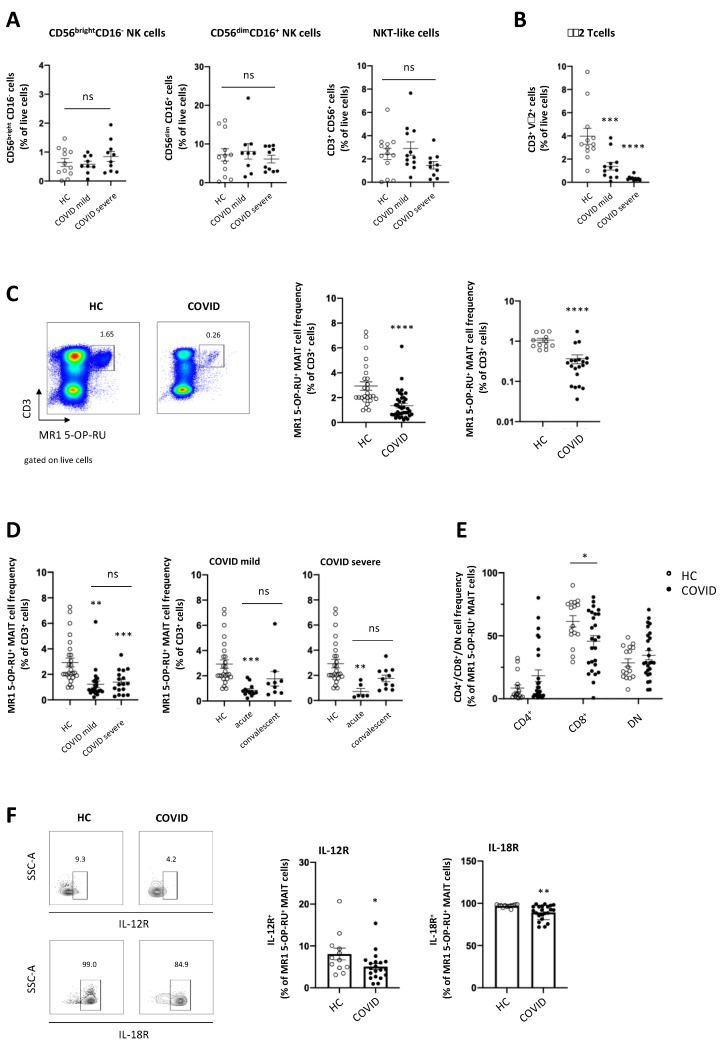
MAIT cells are significantly and continuously reduced in peripheral blood of COVID-19 patients, regardless of disease severity. (**A**) Frequency of natural killer (NK) cells ( HC *n* = 12; COVID mild *n* = 9; COVID severe *n* = 10), NKT-like cells (HC n = 13, COVID mild *n* = 12; COVID severe *n* = 10) and (B) γδ2 T cells (HC *n* = 12; COVID mild *n* = 12; COVID severe *n* = 10) in peripheral blood of COVID-19 patients and healthy controls (HC); (**C**,**D**) mucosal-associated invariant T (MAIT) cell frequency (C: HC *n* = 26 or 12; COVID *n* = 38 or 20; D: HC *n* = 26; COVID mild total *n* = 22, acute *n* = 13, convalescent *n* = 9; COVID severe total *n* = 17, acute *n* = 6, convalescent *n* = 11), (**E**) T cell receptor (TCR) co-receptor expression (HC *n* = 16; COVID *n* = 26) and (**F**) IL-12 receptor (IL-12R): HC *n* = 12; COVID *n* = 20) and IL-18 receptor (IL-18R): HC *n* = 12; COVID *n* = 22) expression in COVID-19 patients and HC. Data are presented as mean ± SEM and were pooled from 3 (**A**,**B**) or >10 (**C**,**D**) independent experiments; each symbol represents one patient; * *p* < 0.05, ** *p* < 0.01, *** *p* < 0.001 and **** *p* < 0.0001 vs. HC or as indicated; data were assessed using one-way analysis of variance (ANOVA) with Tukey’s multiple comparisons test (**A**,**B**,**D**), unpaired t-test (**C**,**F**) or two-way ANOVA with Sidak’s multiple comparisons test (**E**). ns = not significant; DN = CD4/CD8 double-negative.

Besides classical, adaptive T cells, innate natural killer (NK) cells and nonconventional T cells, such as natural killer T (NKT) cells, γδ T cells and MAIT cells, have been proposed as possible important immunological players in COVID-19, since they can rapidly respond to inflammatory signals and orchestrate inflammation [26,27,28]. We therefore analysed the frequency of NK cells and nonconventional T cells in patients with COVID-19 next. As shown in Figure 2A, the frequency of major subsets of NK cells, CD56^bright^CD16^−^ cytokine-producing, and CD56^dim^CD16^+^ cytotoxic NK cells, as well as NKT-like CD3^+^CD56^+^ cells was unchanged in patients with COVID-19 compared to healthy controls, irrespective of the course of disease. In contrast, we observed a significant reduction of γδ2 T cells in both patients with mild and patients with severe COVID-19 (Figure 2B). Along the same line, MAIT cells, which were defined as CD3^+^ MR1 5-OP-RU tetramer^+^ (Figure 2C) or CD3^+^CD161^+^Vα7.2^+^ cells (Figure S2A), were significantly reduced in COVID-19 patients compared to healthy controls (Figure 2C). Since MAIT cells have been shown to be important antimicrobial effectors, we set out to analyse the phenotype and function of MAIT cells in more detail in different subgroups of COVID-19 patients in our cohort. Besides assigning patients according to their clinical course of disease, we subdivided our cohort into acutely infected and convalescent patients. While samples were taken within one week from symptom onset and/or within a maximum of 72 h from hospitalisation in patients assigned to the “acute” group, patients in the “convalescent” group were sampled 4–9 weeks after admission to the hospital. Moreover, SARS-CoV-2 could not be detected anymore by PCR in nasopharyngeal swabs and/or seroconversion, that is, detection of anti-SARS-CoV-2 IgM and/or IgG was documented at the time of sampling in convalescent patients. As shown in Figure 2D, MAIT cells were significantly decreased in both patients with mild and patients with severe COVID-19. Interestingly, such decline of MAIT cells was sustained over time, since MAIT cell frequency did not significantly increase in convalescent patients (Figure 2D). In healthy humans, the majority of MAIT cells are CD8-positive, followed by CD4/CD8 double-negative (DN) MAIT cells [29]. Here, we observed a significant decrease in the proportion of CD8^+^ MAIT cells, while proportions of CD4^+^ MAIT cells and CD4^−^CD8^−^ MAIT cells increased in patients compared to healthy controls, although not significantly (Figure 2E). Such decline of CD8^+^ MAIT cells was observed both in patients with severe and in patients with mild COVID-19 (Figure S2B) and maintained in convalescent patients (Figure S2C). MAIT cells express high levels of cytokine receptors, allowing them for rapid, antigen-independent production of effector cytokines following stimulation with IL-12 and IL-18 [21]. Analysing the expression of the cytokine receptors IL-12R and IL-18R, we observed a significantly lower expression of both IL-12R and IL-18R in MAIT cells isolated from COVID-19 patients compared to healthy controls (Figure 2F). Taken together, these data suggest alterations of the innate and nonconventional T cell compartment in COVID-19. Moreover, our data indicate that MAIT cells are severely reduced in peripheral blood of COVID-19 patients and show phenotypical alterations.

### 3.3. MAIT Cells Express a Highly Activated Phenotype in COVID-19 Patients

**Figure 3:**
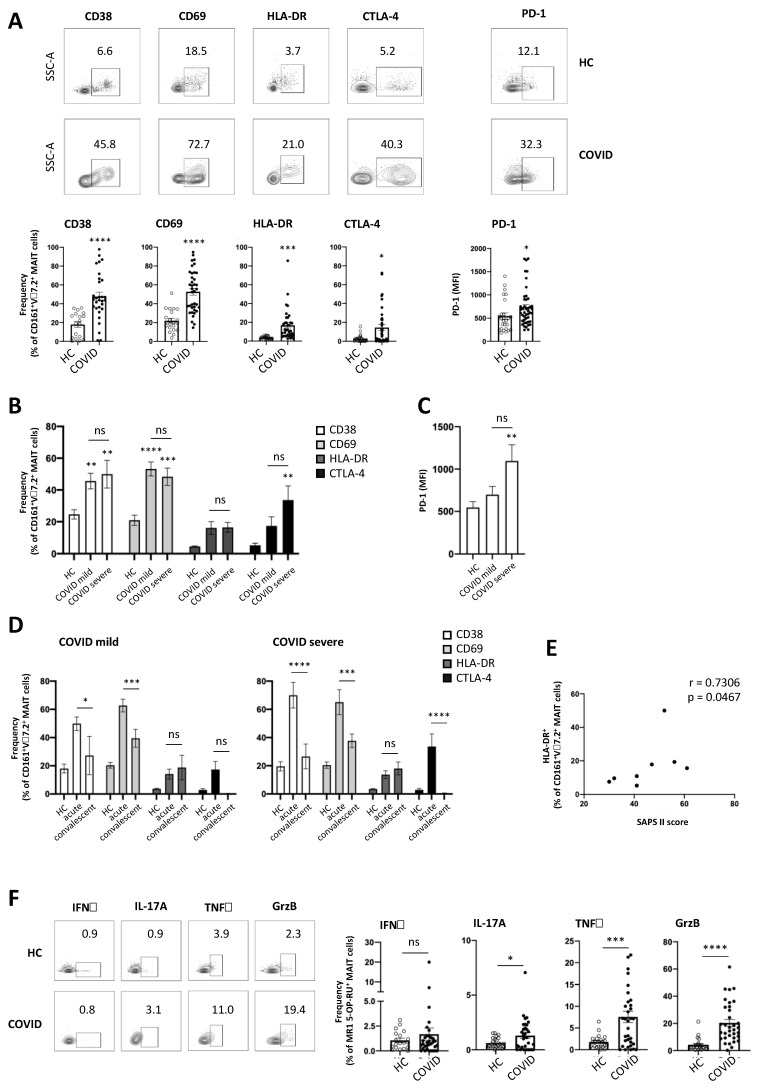
MAIT cells of COVID-19 patients express high levels of activation markers and cytokines ex vivo. (**A**–**D**) Ex vivo expression of surface activation markers in MAIT cells from peripheral blood of COVID-19 patients and healthy controls (HC); (**E**) Spearman correlation between expression of HLA-DR in MAIT cells in peripheral blood of COVID-19 patients and SAPS II score at day of sampling; (**F**) Ex vivo intracellular cytokine expression in MAIT cells from peripheral blood of COVID-19 patients and HC, representative data from one patient and summary data; (**A**,**E**,**F**) each symbol represents one patient. (**A**) CD38: HC *n* = 18 and COVID *n* = 30; CD69: HC *n* = 21 and COVID *n* = 38; HLA-DR: HC *n* = 21 and COVID = 35; CTLA-4: HC *n* = 20 and COVID *n* = 36; PD-1: HC *n* = 23 and COVID *n* = 34. (**B**) HC *n* = 13, COVID mild *n* = 22 and COVID severe *n* = 18; (**C**) HC *n* = 26, COVID mild *n* = 17 and COVID severe *n* = 9; (**D**) HC *n* = 19, COVID acute *n* = 13 and COVID convalescent *n* = 9; (**E**) *n* = 8; (**F**) IFNγ: HC *n* = 19 and COVID *n* = 33; IL-17A: HC *n* = 23 and COVID *n* = 33; TNFα: HC *n* = 23 and COVID *n* = 34; GrzB: HC *n* = 23 and COVID *n* = 34. Data are presented as mean ± SEM and were pooled from six (**A**–**D**) or four (**E**) independent experiments; * *p* < 0.05, ** *p* < 0.01, *** *p* < 0.001 and **** *p* < 0.0001 vs. HC or as indicated. Data were assessed using unpaired t-test (**A**,**F**), two-way analysis of variance (ANOVA) with Tukey’s multiple comparisons test (**B**,**D**) or one-way ANOVA with Tukey’s multiple comparisons test (**C**). ns = not significant.

To investigate the phenotype of MAIT cells in COVID-19 in more detail, we analysed markers of activation and exhaustion in MAIT cells isolated from peripheral blood of COVID-19 patients. As shown in Figure 3A, MAIT cells from COVID-19 patients expressed significantly higher levels of the activation markers CD38, CD69 and HLA-DR, as well as of the exhaustion markers CTLA-4 and PD-1, compared to healthy controls. Of note, expression of HLA-DR and CTLA-4 negatively correlated with MAIT cell frequency in COVID-19 patients (Figure S3A), indicating that the lower frequency of MAIT cells in peripheral blood of COVID-19 patients may result from activation-induced cell death in vivo. When analysing activation marker expression in MAIT cells isolated from patients with mild or severe COVID-19, we observed similar levels of activation marker expression in MAIT cells isolated from both patient groups (Figure 3B), suggesting that MAIT cells are highly activated in COVID-19 patients, even in the absence of severe pulmonary damage and organ failure. Interestingly, expression of exhaustion markers, such as CTLA-4 and PD-1, by MAIT cells was elevated in patients with severe COVID-19, although not significantly (Figure 3B,C). We next analysed MAIT cell activation in convalescent COVID-19 patients that had already cleared SARS-CoV-2 and developed specific antibodies. As shown in Figure 3D, expression of CD38 and CD69 was significantly lower in convalescent patients, irrespective of the course of disease. Along these lines, CTLA-4 expression was significantly lower in convalescent patients with severe COVID-19 and markedly lower in patients with mild COVID-19. In contrast, expression of HLA-DR (Figure 3D) and PD-1 (Figure S3B) showed a tendency to rise in convalescent COVID-19 patients. Moreover, expression of the activation marker HLA-DR positively correlated with SAPS II score (Figure 3E), an indicator of disease severity in patients treated on ICU, in convalescent COVID-19 patients that still required intensive care treatment, hinting towards a role of MAIT cell activation for disease severity. To analyse whether alterations in MAIT cell phenotype in COVID-19 patients were accompanied by functional alterations, we analysed ex vivo cytokine expression in COVID-19 MAIT cells. As shown in Figure 3F, MAIT cells isolated from COVID-19 patients expressed significantly higher levels of the pro-inflammatory cytokines TNFα and IL-17A, as well as of the cytolytic protein granzyme B compared to healthy controls ex vivo, while ex vivo expression of IFNγ and perforin was unchanged (Figure 3F and Figure S3C). We next analysed ex vivo cytokine expression in MAIT cells isolated from patients with mild and severe COVID-19. Interestingly, perforin expression was significantly higher in patients with severe COVID-19 compared with patients with mild COVID-19 and healthy controls, whereas ex vivo expression of IFNγ, TNFα, IL-17A and granzyme B did not differ between MAIT cells isolated from patients with mild or severe COVID-19 (Figure S3D). Taken together, these data suggest that MAIT cells are highly activated in COVID-19 patients.

### 3.4. Expression of Cytokines and Cytolytic Proteins in Response to Specific In Vitro Stimulation is Severely Altered in MAIT Cells from COVID-19 Patients

**Figure 4:**
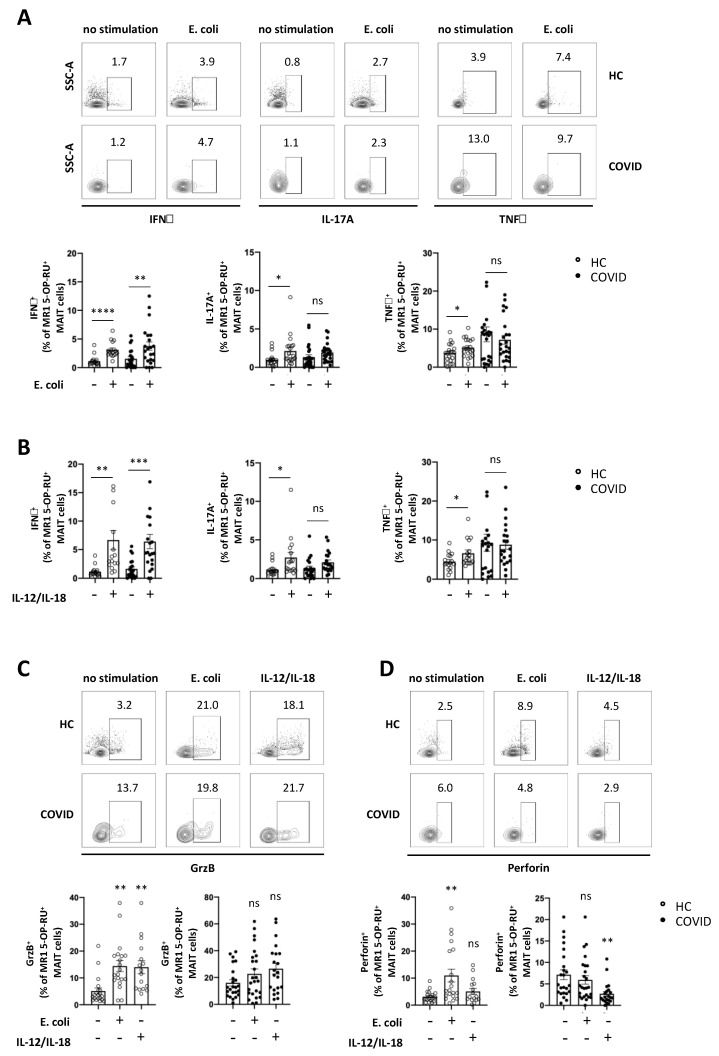
MAIT cells from peripheral blood of COVID-19 patients show an altered cytokine expression profile. Intracellular cytokine expression in MAIT cells in a PBMC pool from peripheral blood of COVID-19 patients and healthy controls (HC) following co-culture with *E. coli* at 10 bacteria per cell (**A**,**C**,**D**) or stimulation with 50 ng/mL IL-12/IL-18 (**B**,**C**,**D**) for 24 h. Data are presented as mean ± SEM and were pooled from three independent experiments; each symbol represents one patient. (**A**) IFNγ: HC *n* = 16 and COVID *n* = 22; IL-17A/TNFα: HC *n* = 20 and COVID *n* = 24; (**B**) IFNγ: HC *n* = 16 and COVID *n* = 21; IL-17A/TNFα: HC *n* = 26 and COVID *n* = 20; (**C**,**D**) HC *n* = 16 and COVID *n* = 21; * *p* < 0.05 and ** *p* < 0.01 vs. unstimulated control. Data were assessed using paired t-test (**A**,**B**) or one-way analysis of variance (ANOVA) with Dunnett’s multiple comparisons test (**C**,**D**). ns = not significant.

To investigate the function of MAIT cells in COVID-19 patients, we analysed MAIT cell response to in vitro stimulation. Since MAIT cells recognise vitamin B metabolites expressed by various strains of bacteria as antigens [11], they can be stimulated in vitro by incubation with Escherichia coli (*E. coli)* [21]. Hence, we incubated PBMCs from COVID-19 patients and healthy controls with paraformaldehyde-fixed *E. coli* for 24 h and analysed intracellular cytokine expression of MAIT cells. As shown in Figure 4A, MAIT cells both from COVID-19 patients and from healthy controls significantly upregulated IFNγ expression following *E. coli* stimulation. In contrast, MAIT cells failed to upregulate expression of IL-17A and TNFα upon *E. coli* stimulation (Figure 4A). Although MAIT cells cannot recognise viral antigen, it has been shown that MAIT cells are activated in viral infections, orchestrate antiviral responses and limit viral replication in vitro [20,22]. Activation of MAIT cells in viral infections is mediated by T cell receptor (TCR)-independent activation by IL-12 and IL-18 [20]. We therefore analysed MAIT cell response to in vitro stimulation with IL-12 and IL-18. Figure 4B and Figure S4A show that MAIT cells from both COVID-19 patients and healthy controls significantly upregulate IFNγ expression upon 24 h stimulation with IL-12 and IL-18. In contrast, upregulation of IL-17A and TNFα was impaired in MAIT cells from COVID-19 patients compared to healthy controls. Next, MAIT cell cytokine expression in response to in vitro stimulation was investigated in MAIT cells isolated from patients with mild or severe COVID-19. We observed a significantly higher IFNγ expression in MAIT cells from patients with severe COVID-19 in response to IL-12/IL-18, but not *E. coli* stimulation, compared with MAIT cells from patients with mild COVID-19 (Figure S4B). In contrast, expression of TNFα and IL-17A was similar in MAIT cells from patients with mild and patients with severe COVID-19, irrespective of the way of stimulation (Figure S4B). It has been shown previously that MAIT cells develop a cytotoxic profile characterised by expression of cytolytic proteins, such as granzyme B and perforin, upon stimulation [14,30], thereby rendering them able to kill bacterially infected cells [14,15,16,17]. We therefore analysed the expression of granzyme B and perforin in MAIT cells from COVID-19 patients and healthy controls upon in vitro stimulation with *E. coli*, or IL-12/IL-18, respectively. While MAIT cells from healthy controls significantly upregulated both granzyme B and perforin expression upon *E. coli* stimulation, MAIT cells from COVID-19 patients expressed high levels of granzyme B and perforin ex vivo (Figure 3F and Figure 4C), but were unable to upregulate expression of both cytolytic proteins upon *E. coli* stimulation (Figure 4C,D). Similarly, MAIT cells from COVID-19 patients failed to upregulate granzyme B expression and downregulated perforin expression upon IL-12/IL-18 stimulation (Figure 4C,D). Solely MAIT cells isolated from patients with severe COVID-19 were able to significantly upregulate granzyme B expression upon IL-12/IL-18 stimulation and expressed significantly higher levels of granzyme B compared to MAIT cells from patients with mild COVID-19 (Figure S4C). Overall, these data suggest that the function of MAIT cells is severely altered in patients with COVID-19 and may result in an impaired antimicrobial capacity.

## 4. Discussion

The immune system, comprising various populations of adaptive and innate immune cells, plays a pivotal role for the pathogenesis of COVID-19. While a functioning immune response is crucial for the elimination of SARS-CoV-2 and thereby resolution of COVID-19, an excessive immune response has been proposed to contribute to the development of severe courses of disease characterised by high mortality [7,9,25,31,32,33]. Therefore, in order to identify effective therapeutic approaches aiming at restoring an adequate function of the immune response, the immunopathogenesis of COVID-19 needs to be recognised in more detail. Here, we demonstrate alterations in phenotype and function of MAIT cells, innate-like T cells, that may contribute to immunopathogenesis in COVID-19. Our data show that MAIT cell frequency is severely reduced in peripheral blood of COVID-19 patients, irrespective of the course of disease, confirming previous reports comparing MAIT cell frequency in COVID-19 patients to a sex- and age-matched control group [25,33,34]. Of note, such MAIT cell decline is sustained even after viral clearance, emphasising that alterations in MAIT cell frequency are profound and long-lasting. It has recently been demonstrated that MAIT cells are enriched in the airways in COVID-19 patients [25,33], suggesting that the observed decline of MAIT cells in peripheral blood results from their recruitment to the inflamed lungs. Murine studies indeed showed that MAIT cells accumulate in the lungs during legionella infection [35], supporting the concept of MAIT cell recruitment to the lungs in COVID-19. However, besides resulting from recruitment to inflamed tissues, MAIT cell decline in COVID-19 may stem from chronic activation in vivo, leading to activation-induced cell death. This is supported by our findings showing that MAIT cells in COVID-19 patients express high levels of activation and exhaustion markers, and that expression of HLA-DR and CTLA-4 negatively correlates with MAIT cell frequency in COVID-19 patients. T cell exhaustion can be induced by various stimuli, for example, chronic exposure to viral antigen [36] or long-term inflammation [37], and is characterised by a hierarchical loss of effector function followed by cell death [38]. We have shown previously that long-term exposure to IL-12 and IL-18 can induce MAIT cell exhaustion [39]. In COVID-19 patients, high serum levels of IL-12 and IL-18 have been described [2,40], further supporting the idea that MAIT cell decline in peripheral blood in COVID-19 patients may, at least partly, result from chronic cytokine exposure in vivo.

Severe COVID-19 is characterised by local and systemic tissue damage resulting in (multi-) organ failure, which often requires intensive care treatment including invasive ventilation or extracorporeal membrane oxygenation [41]. Such tissue damage is thought to result from systemic hyperinflammation, a so-called “cytokine-storm”, that is, uncontrolled immune activation and release of inflammatory cytokines [7,42]. Here, we show that MAIT cells isolated from COVID-19 patients with either mild or severe disease express high levels of activation markers, and that expression levels of such activation markers are highest in acutely infected patients. In line with their innate-like nature, this suggests a contribution of MAIT cells to a rapid, first-line immune response in COVID-19. Moreover, positive correlation of HLA-DR expression with SAPS II, a score indicating disease severity in ICU patients, as well as high ex vivo expression of pro-inflammatory cytokines by MAIT cells in COVID-19 patients further supports the notion of constant in vivo activation of MAIT cells in COVID-19. In addition, one might speculate that MAIT cells thereby contribute to fuelling uncontrolled inflammation and a severe clinical course of COVID-19, which is supported by data showing that MAIT cell activation correlates with a poor clinical outcome in COVID-19 patients [33,34].

Furthermore, to our knowledge, we show for the first time that the ability of MAIT cells from COVID-19 patients to respond to specific, TCR-dependent stimulation, that is, *E. coli,* is impaired in vitro. Even though expression of IFNγ following *E. coli* stimulation is intact, MAIT cells fail to upregulate IL-17 and TNFα, as well as granzyme B and perforin expression upon such stimulation. The inability to further upregulate expression of cytokines and cytolytic proteins upon specific stimulation in combination with high expression of activation and exhaustion markers points towards, at least beginning, functional impairment of MAIT cells in COVID-19 patients. One might even speculate that this hints towards beginning MAIT cell exhaustion due to chronic activation in vivo. MAIT cells are considered important antimicrobial effectors and have been shown to contribute to controlling bacterial infections both by recruitment of other immune cell populations and by lysis of bacterially infected cells [14,15,16,17,43,44]. Our data suggest that the antibacterial effector function of MAIT cells might be impaired in COVID-19. Combined with a possible loss of MAIT cells through activation-induced cell death, such impaired MAIT cell effector function may contribute to a reduced capability to fight bacterial (super-) infections, which are likely to occur in COVID-19 patients [45]. While epidemiological data about the incidence of bacterial superinfections in COVID-19 patients are still missing, there is evidence that the occurrence of bacterial products in COVID-19 patients positively correlates with disease severity [46]. Moreover, the majority of deaths during the influenza pandemic in 1918 were shown to have resulted from secondary bacterial pneumonia [47]. Hence, a functioning antibacterial response represents an important factor contributing to patient morbidity and mortality in COVID-19 patients. When we probed cytokine (i.e. IL-12 and IL-18)-mediated MAIT cell activation, MAIT cells from COVID-19 patients showed an altered cytokine expression profile characterised by impaired upregulation of IL-17A, TNFα, granzyme B and perforin. This suggests that, similar to their antibacterial response, the antiviral response by MAIT cells may be impaired in COVID-19 patients. Although MAIT cells cannot detect viral antigen, it has been shown that MAIT cells are activated in viral infections in a TCR-independent manner requiring signalling through IL-12 and IL-18 [20]. Thus, MAIT cells have been shown to limit viral replication in vitro [20] and to protect against lethal influenza infection in mice [22]. In conclusion, our data suggest that MAIT cell phenotype and function are severely altered in patients with COVID-19 irrespective of the clinical course of disease. The alterations in MAIT cell function observed here may impair the immune response in COVID-19, both by affecting the generation of a functional antimicrobial immune response, as well as by triggering an uncontrolled immune response leading to tissue damage. Taken together, our data contribute to the understanding of the immunopathology in COVID-19 and may facilitate further research towards the development of novel therapeutic strategies.

## Figures and Tables

**Table 1:** Characteristics of coronavirus disease 2019 (COVID-19) patients and healthy controls (HC).

		COVID-19 Mild	COVID-19 Severe	HC
Cases (%)	number	22 (51.2)	21 (48.8)	25
Samples (%)	number	24 (47.1)	27 (52.9)	n.a.
**Baseline Characteristics**				
Age (years)	median	62	65	28
	(range)	(24–86)	(26–84)	(23–41)
Male	number (%)	12 (54.5)	16 (76.2)	13 (52.0)
Female	number (%)	10 (45.5)	5 (23.8)	12 (48.0)
Positive PCR before sampling	number (%)	24 (100)	27 (100)	0 (0)
Symptom onset—sampling (days)	median	9	39.5	n.a.
	(range)	(1–56)	(7–74)	
IL-6 (pg/mL) at sampling	median	12.4	55.7	n.a.
	(range)	(1.5–101.7)	(5.6–11014)	
CRP (mg/dL) at sampling	median	2.8	5.1	n.a.
	(range)	(0.1–10.2)	(0.3–37.3)	
Lymphocyte count total (×10^9^/L)	median	1.0	0.7	n.a.
	(range)	(0.1–2.7)	(0.3–2.4)	
Outcome deceased	number (%)	0 (0)	9 (42,3)	n.a.
**Oxygen Supply**				n.a.
Oxygen therapy <15L	number (%)	6 (27.3)	2 (9.5)	
Noninvasive ventilation	number (%)	0 (0)	0 (0)	
Invasive ventilation	number (%)	0 (0)	19 (90.5)	
ECMO	number (%)	0 (0)	5 (23.8)	
**Comorbidities**				
No comorbidity	number (%)	5 (22.7)	1 (4.8)	25 (100.0)
Hypertension	number (%)	9 (40.9)	14 (66.7)	
Diabetes	number (%)	4 (18.2)	8 (38.1)	
Coronary heart disease	number (%)	2 (9.1)	4 (19.0)	
COPD/Asthma	number (%)	4 (18.2)	1 (4.8)	
Chronic kidney disease	number (%)	0 (0)	6 (28.6)	
Cancer (under treatment)	number (%)	4 (18.2)	5 (23.8)	
**Disease Severity (ICU)**				n.a.
SOFA at admission to ICU	median	n.a.	11	
	(range)		(2–14)	
SAPS II at sampling	median	n.a.	41	
	(range)		(13–61)	
**COVID-Specific Treatment**				n.a.
Treatment total	number (%)	5 (22.7)	15 (71.4)	
- Dexamethason	number (%)	0 (0)	8 (38.1)	
- Remdesivir	number (%)	5 (22.7)	6 (28.6)	
- Convalesent plasma	number (%)	1 (4.5)	3 (14.3)	
- Hydrocortison	number (%)	0 (0)	5 (23.8)	

n.a. = not applicable.

## Data Availability

The data presented in this study are available in “Mucosal-associated invariant T (MAIT) cells are highly activated and functionally impaired in patients with COVID-19”.

## Supplementary Materials

The following are available online at https://www.mdpi.com/1999-4915/13/2/241/s1. Figure S1: Gating strategy for flow cytometry. Figure S2: MAIT cell activation and ex vivo cytokine expression. Figure S3: Cytokine expression of MAIT cells following in vitro stimulation; Figure S4: Cytokine expression of MAIT cells following in vitro stimulation Table S1: Characteristics of acute and convalescent COVID-19 patients.

## Abbreviations

ARDS	acute respiratory distress syndrome
CD	cluster of differentiation
COPD	chronic obstructive pulmonary disease
COVID-19	coronavirus disease 2019
CRP	C-reactive protein
CTLA-4	cytotoxic T-lymphocyte-associated protein 4
*E. coli*	*Escherichia coli*
ECMO	extracorporeal membrane oxygenation
HLA-DR	human leukocyte antigen—DR isotype
ICU	intensive care unit
IFNγ	interferon γ
IL	interleukin
MAIT cells	mucosal-associated invariant T cells
NK cells	natural killer cells
PBMCs	peripheral blood mononuclear cells
PCR	polymerase chain reaction
PD-1	programmed cell death protein 1
SAPS	Simplified Acute Physiology Score
SARS-CoV-2	severe acute respiratory syndrome coronavirus 2
SIRS	systemic inflammatory response syndrome
SOFA	sepsis-related organ failure assessment
TNFα	tumour necrosis factor α
WHO	World Health Organisation
