# Supplementary material for: Mucosal-Associated Invariant T (MAIT) Cells Are Highly Activated and Functionally Impaired in COVID-19 Patients
Source: Viruses. 2021 Feb 3;13(2):241. doi: 10.3390/v13020241 (PMC7913667; doi:10.3390/v13020241)
Supplement: Supplementary file 1 [file viruses-13-00241-s001.pdf]

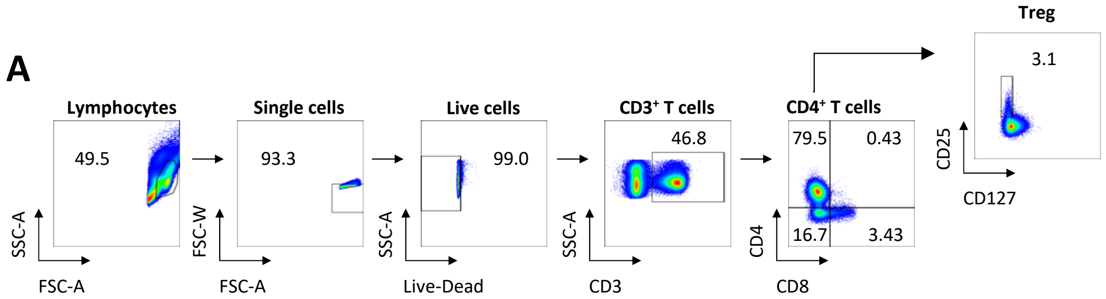

Figure S1. Gating strategy for flow cytometry.Gating strategy for regulatory T cells (Treg), defined as CD3$^+$CD4$^+$CD25$^+$CD127$^-$ cells.

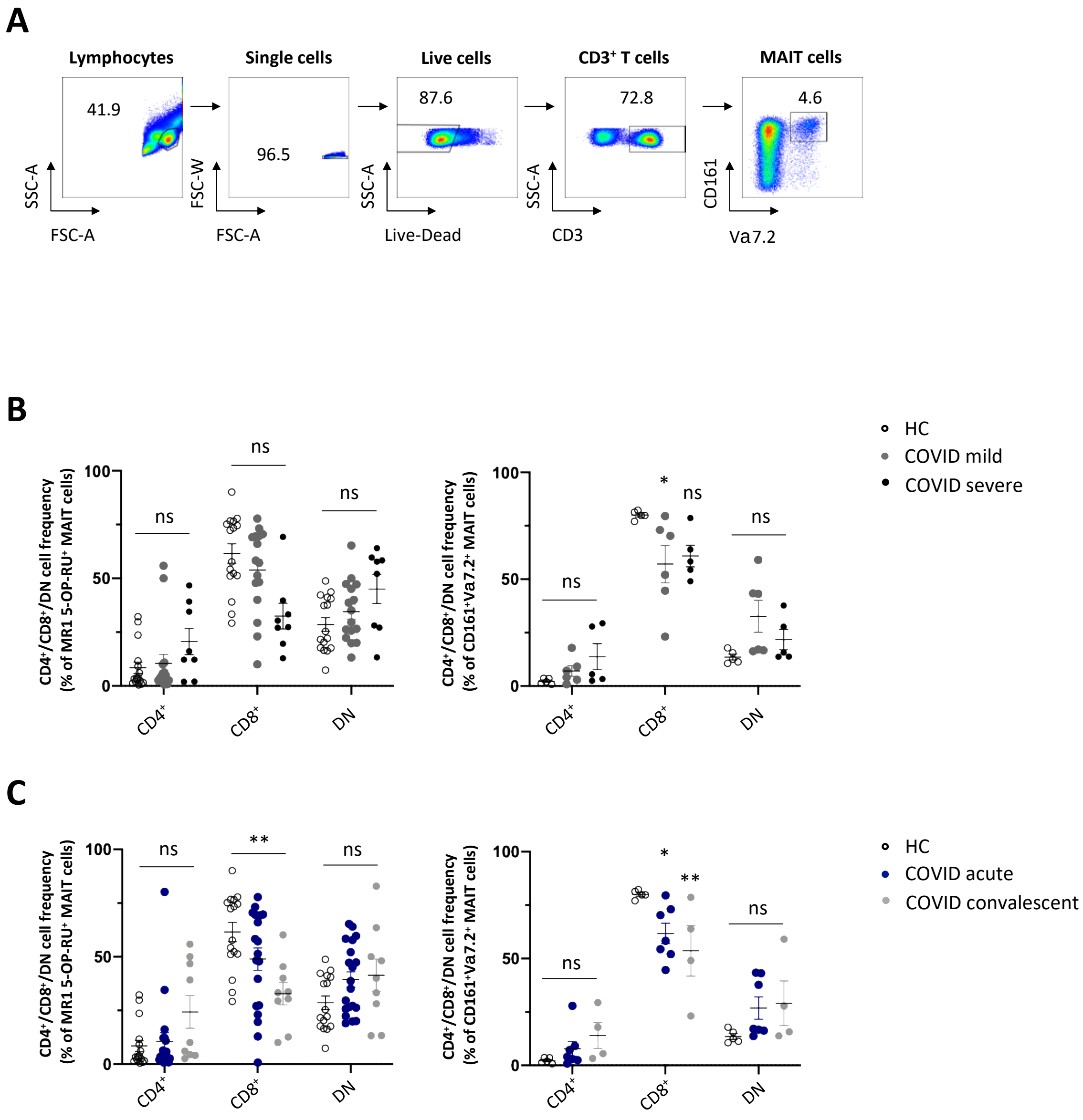

Figure S2. MAIT cell gating strategy and T cell receptor (TCR) co-receptor expression. (**A**) Gating strategy for MAIT cells, defined as CD3$^+$CD161$^+$ Va7.2$^+$ cells. (**B**,**C**) TCR co-receptor expression in CD3$^+$MR1 5-OP-RU$^+$ and CD3$^+$CD161$^+$Va7.2$^+$ MAIT cells isolated from peripheral blood of COVID-19 patients and healthy controls (HC), HC n = 16/5, COVID mild n = 16/7, COVID severe n = 8/5, (C) HC n = 16/5, COVID acute n = 20/7, COVID convalescent n = 9/4. Data are presented as mean ± SEM and were pooled from 5 independent experiments; each symbol represents one patient; * $p < 0.05$, ** $p < 0.01$ vs. healthy control or as indicated; data were assessed using

two-way analysis of variance (ANOVA) with Tukey's multiple comparisons test (**B**,**C**); ns = not significant, DN = CD4/CD8 double negative.

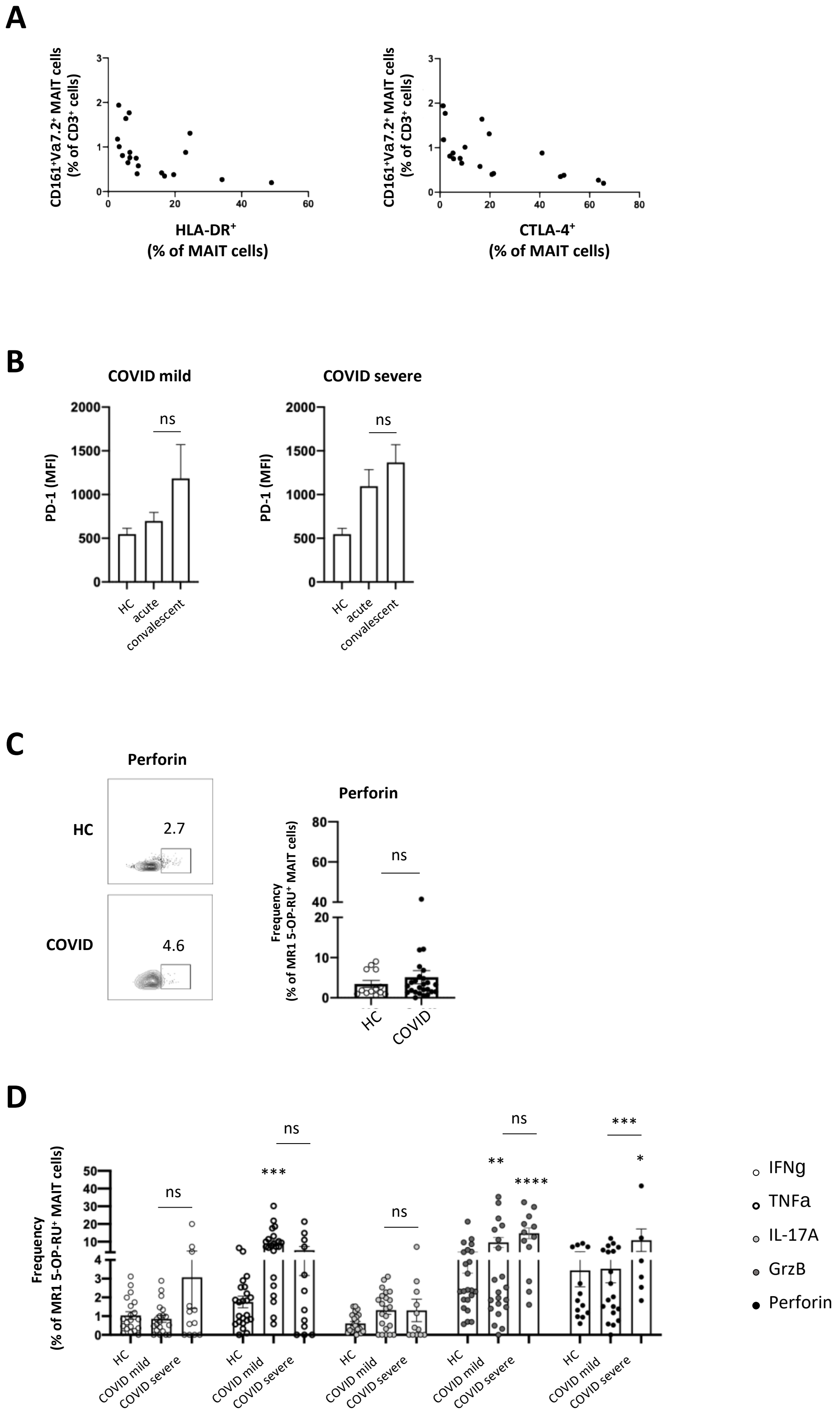

Figure S3. MAIT cell activation and ex vivo cytokine expression. (**A**) Spearman correlation of MAIT cell frequency in peripheral blood of COVID-19 patients and expression of activation and exhaustion markers, *n* = 19. (**B**) PD-1 MFI in $CD3^{+}CD161^{+}Va7.2^{+}$ MAIT cells isolated from peripheral blood of acute and convalescent COVID-19 patients

and healthy controls (HC), HC $n = 26$, COVID acute $n = 9$, COVID convalescent $n = 6$. (**C**,**D**) Ex vivo intracellular cytokine expression in $CD3^{+}$MR1 5-OP-RU^{+} MAIT cells from peripheral blood of COVID-19 patients and healthy controls, (**C**) HC $n = 13$, COVID $n = 25$, (D) HC $n \geq 13$, COVID mild $n \geq 19$, COVID severe $n \geq 6$. Data are presented as mean +/- SEM and were pooled from 3 independent experiments; (**A**,**C**,**D**) each symbol represents one patient,; * $p < 0.05$, ** $p < 0.01$, *** $p < 0.001$, **** $p < 0.0001$ vs. HC or as indicated, data were assessed using one-way analysis of variance (ANOVA) with Tukey's multiple comparisons test (**B**), unpaired t-test (**C**) or two-way ANOVA with Tukey's multiple comparisons test (**D**); ns = not significant

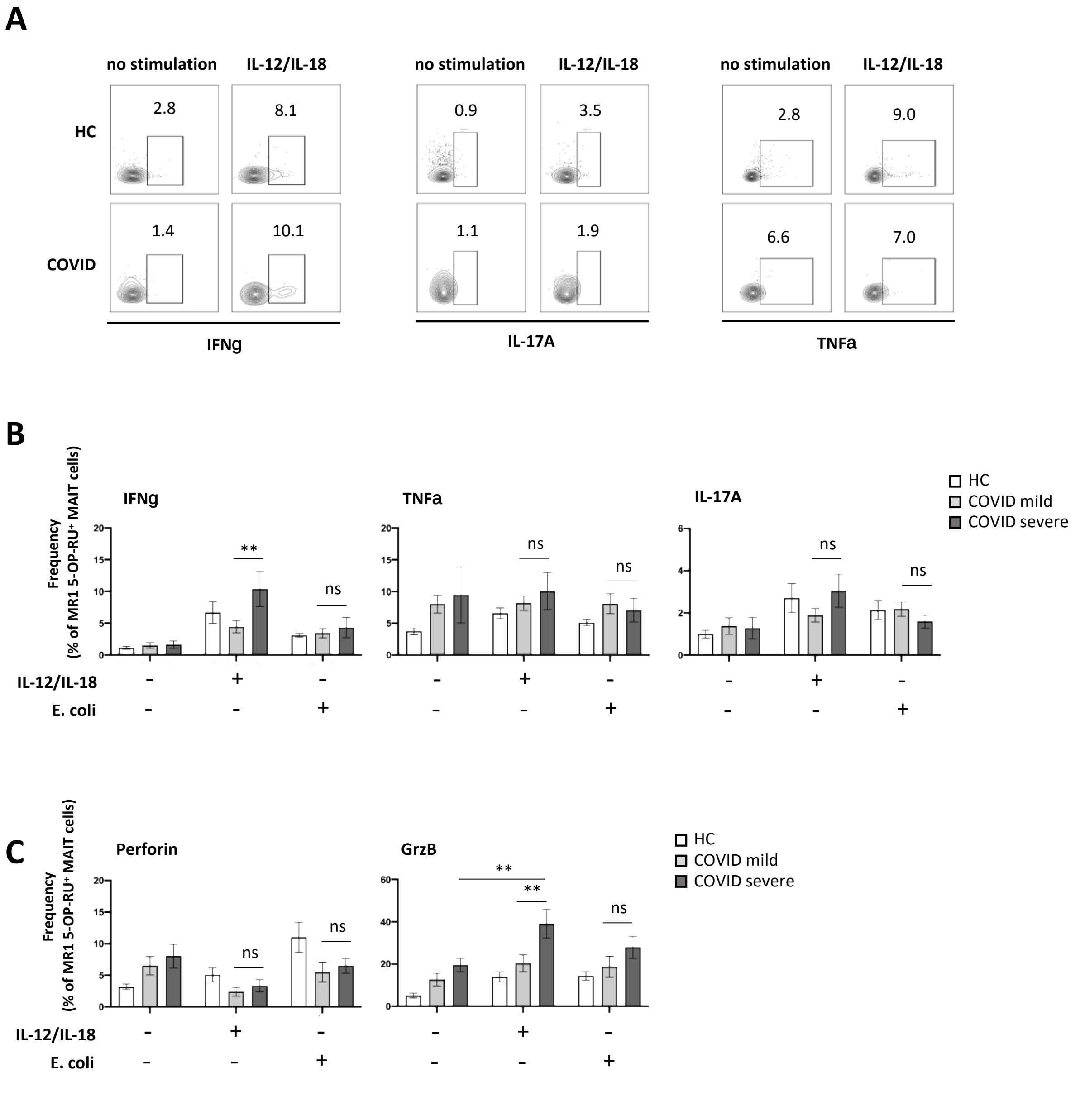

Figure S4. Cytokine expression of MAIT cells following in vitro stimulation. Intracellular cytokine expression in $CD3^{+}$MR1 5-OP-RU^{+} MAIT cells from COVID-19 patients and healthy controls (HC) following stimulation with 50 ng/ml IL-12/IL-18 (**A**–**C**) or co-culture with E. coli at 10 bacteria per cell (**B**,**C**) for 24 hours. Data are presented as mean ± SEM and were pooled from 3 independent experiments; HC $n = 15$, COVID mild $n = 15$, COVID severe $n = 15$; ** $p < 0.01$, data were assessed using two-way analysis of variance (ANOVA) with Tukey's multiple comparisons test; ns = not significant.

Table S1. Characteristics of acute and convalescent COVID-19 patients.

		**COVID-19 acute**	**COVID-19 convalesent**
Cases	number	23	19
Samples	number	24	26
Baseline characteristics			
Age (years)	median	66	61
	(range)	(26-86)	(24–84)
Male	number (%)	14	13 (68.4)
Female	number (%)	9	6 (31.6)
Infectious diseases ward	number (%)	15	7 (36.8)
Intensive care unit	number (%)	8	12 (63.2)
Symptom onset - sampling (days)	median	8	45.5
	(range)	(1-21)	(9-74)
